# Abstract of Proceedings of the Buffalo Medical Association

**Published:** 1869-02

**Authors:** T. M. Johnson

**Affiliations:** Sec’y.


ART. IV.—*Abstract of Proceedings of the Buffalo Medical Association.*

TUESDAY EVENING, January 5th, 1869.

The President and Vice President being absent, Dr. Gay was elected chairman of the meeting.

Members present—Drs. Gay, Ring, Cronyn, Samo, Trowbridge, Diehl, White, Hauenstein and Johnson. The minutes of the last meeting were approved.

DR. T. M. JOHNSON reported the following case of rheumatism: Was called a few weeks ago to see a child then but five days old, which was taken on the morning previous with pain and restlessness, and later in the day with swelling in the right hip, and when first seen by me the right hip and right knee were painful, red and swollen. On the day following the local symptoms had extended to the opposite knee and ankle, but had partially subsided at the point of first attack. There was the usual febrile excitement and other phenomena of acute rheumatism, which continued for four days, when death came to the relief of the little sufferer.

The father of this child had suffered with rheumatism for nearly a year, and until within three months previous to the birth of the child. The grandfather of the child had also had rheumatism. The mother died three days after the birth of the child of pyæmia. The doctor said that he was satisfied that there was no syphilitic taint in the case, but that it was an uncomplicated case of rheumatism, and probably congenital.

DR. CRONYN remarked that he had been very much interested in the case, having never known rheumatism to attack so young a child. He believed that the disease was congenital, and believed that it might, like gout, be transmitted from parent to child.

DR. J. S. TROWBRIDGE said that it had several times been intimated to him by members of the Historical Society that the Society was desirous of having the portrait of Dr. Josiah Trowbridge, now in our possession, transferred to the possession and rooms of their Society. The doctor said that he introduced the matter not with a view to influence any action the Association might take in the matter, but simply to fulfill an obligation to the persons before mentioned.

This matter was discussed by all the members present, none of whom were in favor of the transfer. The sense of those present will be seen by the following resolution, which was passed unanimously:

*Resolved*, That the President appoint a committee of five to obtain portraits of the ex-Presidents of this Association and eminent deceased members of the profession of Buffalo.

The President appointed the following gentlemen a committee: Drs. J. S. Trowbridge, James P. White, J. R. Lothrop, Thomas M. Johnson and John Cronyn.

Dr. Hauenstein reported the following case of Spurious Melanosis, and said that this was the first case that he had ever seen in which *post mortem* examination proved the disease to be spurious melanosis. This patient, Mr. Knapf, was a man of fifty years of age, and a moulder by trade. Saw him for the first time on December 1st, and found him complaining of extreme pain in the left side. I learned at this time that about three months ago he received a severe blow upon the middle third of the sternum. The effects of the blow were quite severely felt, but he continued his usual labor. About three weeks before I first saw him he was taken with considerable cough, but he continued work until the day before my first call. I did not determine at this time whether it was pleurisy or neuralgia. Discovered no friction sound. To relieve pain seemed the indication; this was done by hypodermic injection of morphia. The second day found pneumonia existing. On the seventh day I considered him convalescing and prognosticated good results. On the eighth day, however, he had a severe chill with high pulse, followed with a paroxysm of coughing, after which the breath was quited fetid. I thought there might be abscess, but the fetor soon disappeared. I called Dr. J. R. Lothrop to see the case with me; the doctor diagnosed pleuritis with effusion. The treatment recommended was iodide of potassa with opiates. Two days after this Dr. Rochester saw the patient with me and concurred with my views, and we thought it was nothing but pleuritis. The following day I was discharged and Dr. Toby was called. Four or five days after Dr. Toby first saw him he expectorated three pints of black fetid matter, then seemed better for a day, and then expectorated as before, and died about three weeks after Dr. Toby was first called.

*Post mortem* examination showed the lungs to be as black as ordinary carbon. They were taken from the thorax, and the left lung was adhered to the walls of the thorax and to the diaphragm throughout their whole extent. The right lung was not so much adhered as the left. The superior lobe of the right lung was of the black color. The left lung was nearly impervious. The pericardium contained seven ounces of fluid similar to that expectorated. The heart was a little less in size than normal. The liver was slightly enlarged. Kidneys and spleen healthy. The coroner's jury in the case decided in their minds that the blow did not produce the disease or the death.

Dr. White said, I do not feel competent to speak upon this subject, never having seen more than three cases, and those in consultation. I cannot conceive that the blow had anything to do with the development of the disease. The first case that ever came under my notice there was abscess of the liver and vomiting of bile. This was a case of melanosis of the liver.

Scarlatina, pneumonia and influenza, were reported as the prevailing diseases. Adjourned.

T. M. Johnson, Sec'y.

